# Age-related sex differences in intensive care treatment and outcomes: a nationwide cohort study

**DOI:** 10.1016/j.bja.2025.07.044

**Published:** 2025-08-29

**Authors:** Elsa Hägglöf, Jesper Eriksson, Max Bell, Linn Hallqvist, Lars Engerström, Emma Larsson

**Affiliations:** 1 Department of Physiology and Pharmacology, Karolinska Institutet, Solna, Sweden; 2 Department of Perioperative Medicine and Intensive Care, Karolinska University Hospital, Stockholm, Sweden; 3 Department of Cardiothoracic and Vascular Surgery and Anaesthesia, and Department of Medical and Health Sciences Linköping University Hospital, Linköping, Sweden; 4 Department of Anaesthesia and Intensive Care Medicine, and Department of Biomedical and Clinical Sciences, Vrinnevi Hospital, Norrköping, Sweden

**Keywords:** age factors, critical care, gender, intensive care, mortality, sex

## Abstract

**Background:**

Reported differences in treatment and mortality between male and female patients in the ICU are inconsistent. Previous studies suggest that age might influence sex differences, partly explaining earlier discrepancies. This study aimed to compare patient characteristics, care intensity, and mortality between male and female ICU patients while examining potential age-varying sex differences.

**Methods:**

All adult (≥18 yr) ICU patients between 2012 and 2024 were identified in the Swedish Intensive Care Registry. Multivariable logistic regression models, adjusted for age and Simplified Acute Physiology Score 3, investigated associations between patient sex and 30-day mortality, continuous renal replacement therapy, and invasive ventilation. Analyses included the entire cohort and six diagnostic subgroups. To describe the varying effect of sex across ages, a multivariable logistic regression model, using natural cubic splines, allowed age to interact with sex.

**Results:**

We included 303 875 ICU patients (median [interquartile range] age: 67 [51–76] yr; 42.8% female). Crude mortality was higher in male patients (odds ratio [OR] 0.94, confidence interval [CI] 0.92–0.95). In multivariable models, female sex was associated with higher 30-day mortality (OR 1.03, CI 1.01–1.05). Male patients were more likely to receive continuous renal replacement therapy and invasive ventilation. Female patients had lower mortality risk at younger ages and higher mortality risk at older ages.

**Conclusions:**

Although males constitute a larger proportion of ICU patients and receive more advanced treatments, females have higher adjusted mortality. This is nuanced by age-specific variations, which underscore the complexity and necessity of considering age when evaluating sex-based differences in ICU outcomes.

**Editor’s key points**

• The effect of patient sex on admission, treatment, and outcomes in ICU is an area of active study. However, the intersectionality between sex and age requires further investigation.
• Sweden is fortunate to have has a national registry including individual patient data from all its 81 ICUs. The authors used this registry to investigate whether patient characteristics, care intensity, and mortality vary between male and female ICU patients, and to examine potentially age-varying sex differences.
• Crude 30-day mortality was higher in males than in females. However, after adjustment for age and illness severity, female sex was associated with higher 30-day mortality. Males were more likely to receive continuous renal replacement therapy and invasive ventilation. Females experienced lower mortality risk at younger ages and higher mortality risk at older ages.
• Further investigation into underlying mechanisms, including hormonal differences, severity score calibration, and limitations of treatment, is required.

Despite patient sex not being an established factor for determining treatment strategy or approximating risk of mortality, intensive care can differ between male and female patients. Studies have consistently shown a skewed distribution of ICU admissions in favour of male patients.^1^^,^^2^ This contrasts with the older population in society, which is normally dominated by women owing to their longer life expectancy.^3^ Studies have reported that female patients have lower ICU admission probability regardless of disease severity,^4^ receive less treatment,^5^^,^^6^ and spend fewer days in the ICU.^6^ Differing treatment intensity has been considered reasonable, as results regarding sex differences in mortality after intensive care have been conflicting.^7^^,^^8^ Various explanations for greater need for intensive care in males have been proposed, including a potential protective effect of oestrogen^9^ and varying susceptibility to infection.^10^

As the evidence of differing overall ICU mortality between male and female patients has been inconsistent, studies have investigated subgroups. Mortality disparities in the ICU were widely investigated during the COVID-19 pandemic as male sex was associated with higher mortality.^11^ As oestrogen has been central to the issue of differences in biological conditions between males and females, premenopausal age has also been compared with postmenopausal age but without being able to demonstrate an advantage for premenopausal females.^1^ Studies have indicated an increased mortality in female patients compared with male patients among older patients after intensive care.^2^^,^^12^ However, there is a lack of studies exploring whether the influence of patient sex fluctuates with age. The aim of this registry-based cohort study was to investigate differences in patient characteristics, care intensity, and mortality between male and female ICU patients while closely examining potential age-varying sex differences.

## Methods

The cohort study was approved by the Swedish Ethical Review Authority (2023-05595-01 and 2023-07554-02). The Swedish Intensive Care Registry (‘the Registry’) collects individual patient data from all Swedish ICUs (*n*=81) and operates within the legal framework of the Swedish National Quality Registries which does not require written informed consent from the patients, although patients may withdraw their data from the registry at any time. The Registry was the sole source of data for this study.

### Eligibility criteria

We identified all adult (≥18 yr) patients in Swedish ICUs between January 1, 2012 and August 15, 2024 who were registered in the Registry. Exclusion criteria were concealed or temporary personal identity number, invalid registration date of death, emigration within 30 days after admission, and missing Simplified Acute Physiology Score 3 (SAPS3). If multiple admissions of a single patient were present in the dataset, only the first admission was included.

### Covariates and outcomes

Baseline characteristics such as age and sex were defined at time of admission to the ICU. SAPS3 was used to describe illness severity at admission. Total SAPS3 calculates the estimated mortality risk (EMR). Both the global version of EMR, corresponding to in-hospital mortality, and the Swedish calibration, corresponding to 30-day mortality,^13^ were collected.

Comorbidity data in SAPS3 included metastatic cancer, cancer therapies (e.g. chemotherapy, immunosuppression, radiotherapy, steroid treatments), chronic heart failure (New York Heart Association IV), haematologic cancer, cirrhosis, acute lower respiratory infection, and nosocomial infection. SAPS3 also captured location before ICU admission and allowed for multiple predefined reasons for ICU admission. At discharge, each patient was assigned one primary diagnosis as ICD-10 code, which was used to define five subgroups: cardiac arrest, acute respiratory distress syndrome (ARDS), bacterial pneumonia, trauma, and acute brain injury (Supplementary Table 5). A sixth group, the sepsis subgroup, was identified using primary or secondary diagnoses, as sepsis is not a primary diagnosis in the Registry since 2018. Patients could therefore belong to both a primary diagnosis subgroup and the sepsis group. The cohort was also divided into two age groups based on median menopausal age in Sweden (premenopausal <51 yr, postmenopausal ≥51 yr).

The Registry contains data on performed interventions. The outcomes continuous renal replacement therapy (CRRT) and invasive ventilation were defined as presence of the intervention at least once during an admission. To estimate the use of resources in the ICU, the variables *Vårdtyngd Sverige 2014* (VTS2014) and ICU length of stay were used. VTS2014 measures the workload of each admission in Swedish ICUs and comprises 11 indicators, which together yield a maximum of 99 points per day. VTS2014 was presented as a total score for the admission and as score per hour of admission.

Follow-up data on mortality are available in the Registry owing to the Swedish personal identity number, which is assigned to all citizens at birth or upon immigration to Sweden. It enables the Registry to continuously retrieve survival and mortality data from the Swedish Cause of Death Register. Data on mortality can be missing for several reasons, mainly concealed identity, a temporary personal identity number, or emigration within 30 days of ICU admission. 30-day mortality was defined as death within 30 days from ICU admission.

The variable patient ‘sex’ was defined by ‘legal gender’, which is represented as a number within the Swedish identity number. Legal gender in Sweden is binary (male or female). Although it is possible to change one's legal gender in Sweden, it remains an uncommon occurrence^14^ and the variable primarily reflects sex assigned at birth. Therefore, the variable was referred to as sex in this study.

The primary outcome was 30-day mortality. Secondary outcomes were treatment with CRRT or invasive ventilation. The aim was to investigate associations between patient sex and outcomes.

### Statistical methods

Categorical variables were presented as numbers and percentages, continuous variables without approximately symmetric distributions were presented as medians and interquartile range (IQR). Logistic regression assessed associations between sex and 30-day mortality. Univariable models showed crude associations, whereas multivariable models adjusted for age and SAPS3 score (with age subtracted to avoid collinearity) to compare females and males at equal conditions. Similar models analysed CRRT and invasive ventilation. Subgroup analysis was carried forward in the six prespecified diagnostic subgroups and in the age groups.

Two additional analyses were carried forward to ensure robust findings. In the first analysis, patients admitted between March 11, 2020 and September 27, 2021 were excluded to account for healthcare disruptions during COVID-19. These dates mark the WHO's pandemic declaration and the lifting of most restrictions in Sweden. In the second analysis, a mixed-effects model incorporated hospital site as a random intercept, accounting for potential variability across sites.

To examine the age-varying effect of sex, nonlinear terms for age were included to allow for a nonlinear relationship between age and the odds of 30-day mortality. A logistic regression model with natural cubic splines for age, interacting with sex (splined age∗sex), was fitted, adjusting for SAPS3. Knots were placed per Harrell's recommendations when using three to seven knots^15^ and then selecting three knots (10th, 50th, and 90th percentiles) based on Bayesian Information Criterion. The interaction model without splines, the splined model without interaction, and the final model were compared with the original model using the likelihood ratio test. Similar analyses were conducted for diagnostic subgroups and the outcomes CRRT and invasive ventilation. For each age, odds ratios (OR) and 95% confidence intervals (CIs) were calculated for both sexes with SAPS3 fixed at its mean. The marginal OR comparing females with males was plotted.

Analyses used R software (Version 4.1.1, R Foundation for Statistical Computing, Vienna, Austria).^16^ All tests were two-tailed and a *P*-value <0.05 was considered statistically significant.

## Results

**Fig 1:**
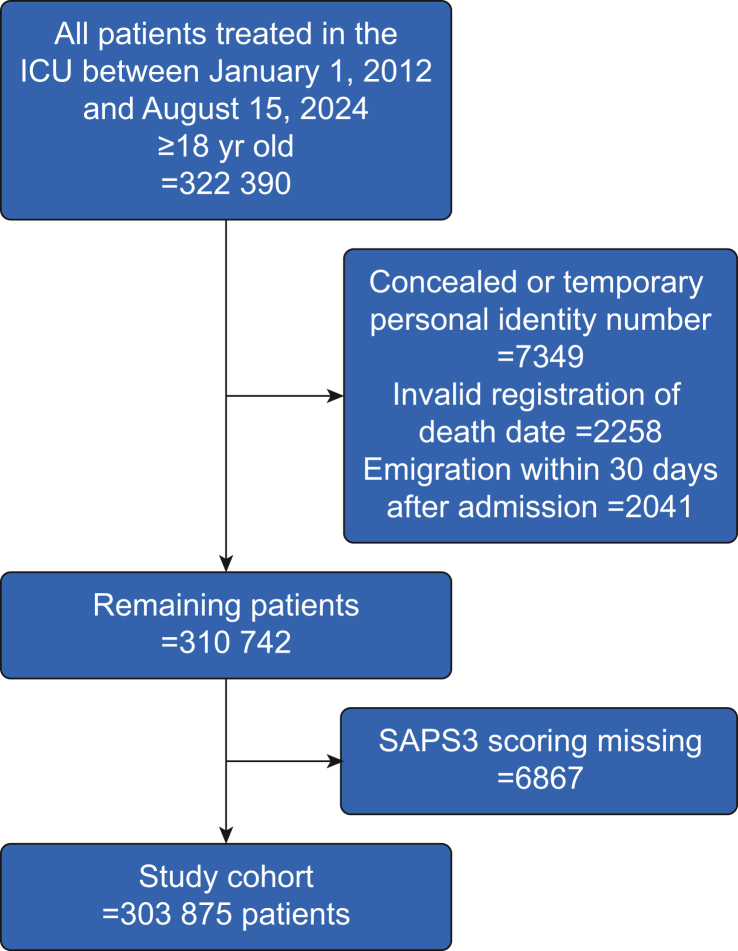
Flow chart of included patients. SAPS3, Simplified Acute Physiology Score 3.

In total, 303 875 patients were included in the study cohort after excluding patients who could not be followed up (Fig. 1). Characteristics of the full cohort are presented in Table 1. Median (IQR) age was 67 (51–76) yr and 42.8% of the patients were female.

**Table 1:** Patient characteristics. CRRT, continuous renal replacement; IQR, interquartile range; SAPS3, Simplified Acute Physiology Score 3; VTS2014, Vårdtyngd Sverige 2014. ∗Chemotherapy, immunosuppression, radiotherapy, steroid treatment. ^†^New York Heart Association IV. Patients can be assigned several SAPS3 reasons for admission.

	Female	Male	Overall
Included patients, *n* (%)	130 011 (42.8)	173 864 (57.2)	303 875 (100)
Age (yr), median (IQR)	67 (49–77)	67 (52–76)	67 (51–76)
**Age group, *n* (%)**			
Premenopausal (<51 yr)	34 261 (45.7)	40 770 (54.3)	75 031 (100)
Postmenopausal (≥51 yr)	95 750 (41.8)	133 094 (58.2)	228 844 (100)
SAPS3 score, median (IQR)	54 (43–65)	55 (44–67)	54 (44–66)
Estimated mortality risk (Swedish), median (IQR)	0.11 (0.03–0.28)	0.12 (0.04–0.32)	0.11 (0.04–0.30)
Estimated mortality risk (Original), median (IQR)	0.24 (0.09–0.46)	0.26 (0.10–0.50)	0.24 (0.10–0.48)
**Location before ICU admission, *n* (%)**			
Emergency department	67 016 (51.5)	91 744 (52.8)	158 760 (52.2)
Hospital floor	38 882 (29.9)	49 860 (28.7)	88 742 (29.2)
**Comorbidity in SAPS3, *n* (%)**			
Metastatic cancer	10 769 (8.3)	15 101 (8.7)	25 870 (8.5)
Cancer therapy∗	7090 (5.5)	8379 (4.8)	15 469 (5.1)
Chronic heart failure^†^	6619 (5.1)	10 513 (6.0)	17 132 (5.6)
Haematologic cancer	1930 (1.5)	3392 (2.0)	5322 (1.8)
Cirrhosis	2205 (1.7)	3973 (2.3)	6178 (2.0)
Acute lower respiratory infection	15 920 (12.2)	25 197 (14.5)	41 117 (13.5)
Nosocomial infection	3348 (2.6)	5046 (2.9)	8394 (2.8)
**Reason for admission in SAPS3, *n* (%)**			
Observation	15 664 (12.0)	18 655 (10.7)	34 319 (11.3)
Cardiovascular	43 822 (33.7)	61 735 (35.5)	105 557 (34.7)
Hepatic	5458 (4.2)	7473 (4.3)	12 931 (4.3)
Gastrointestinal	15 765 (12.1)	21 371 (12.3)	37 136 (12.2)
Neurological	43 173 (33.2)	58 086 (33.4)	101 259 (33.3)
Renal	17 505 (13.5)	27 339 (15.7)	44 844 (14.8)
Respiratory	43 354 (33.3)	60 079 (34.6)	103 433 (34.0)
Haematologic	5289 (4.1)	6657 (3.8)	11 946 (3.9)
Metabolic	28 851 (22.2)	33 897 (19.5)	62 748 (20.6)
Trauma	7748 (6.0)	19 526 (11.2)	27 274 (9.0)
Other	14 544 (11.2)	17 494 (10.1)	32 038 (10.5)
**Diagnostic subgroup, *n* (%)**			
Number with data	129 620 (99.7)	173 360 (99.7)	302 980 (99.7)
Cardiac arrest	5680 (4.4)	11 156 (6.4)	16 836 (5.6)
ARDS	1197 (0.9)	2141 (1.2)	3338 (1.1)
Bacterial pneumonia	3500 (2.7)	5114 (2.9)	8614 (2.8)
Trauma	2455 (1.9)	7279 (4.2)	9734 (3.2)
Acute brain injury	9081 (7.0)	10 905 (6.3)	19 986 (6.6)
Number with data	129 748 (99.8)	173 512 (99.8)	303 612 (99.8)
Sepsis	17 031 (13.1)	23 149 (13.3)	40 180 (13.2)
Length of ICU stay, median (IQR)	24.4 (13.0–55.3)	26.3 (13.3–67.1)	25.4 (13.2–62.8)
**ICU workload**			
Number with data, *n* (%)	83 285 (64.1)	112 630 (64.8)	195 915 (64.5)
VTS2014 score, median (IQR)	62 (34–131)	68 (36–156)	65 (35–145)
VTS2014 per hour admitted to the ICU, median (IQR)	2.44 (2.03–3.05)	2.46 (2.06–3.07)	2.45 (2.05–3.06)
**Interventions in the ICU, *n* (%)**			
CRRT	5008 (3.9)	8721 (5.0)	13 729 (4.5)
Invasive mechanical ventilation	40 862 (31.4)	62 714 (36.1)	103 576 (34.1)
**Mortality, *n* (%)**			
ICU mortality	12 329 (9.5)	17 666 (10.2)	29 995 (9.9)
30-day morality	25 393 (19.5)	35 806 (20.6)	61 199 (20.1)

### Mortality

Overall, 30-day mortality was 20.1% (19.5% in females and 20.6% in males; Table 1). In univariable analysis of the full cohort, female sex was associated with reduced 30-day mortality compared with male sex (OR 0.94, 95% CI 0.92–0.95), but when adjusting for age and SAPS3, female sex was associated with increased 30-day mortality (OR 1.03, 95% CI 1.01–1.05; Table 2). The results were consistent when excluding the COVID-19 period (Supplementary Table 1) and when clustering by site (Supplementary Table 2). When adjusting for only age, results for the full cohort were similar to those observed in the univariable analysis (Supplementary Table 3). In the diagnostic subgroups, female sex was associated with increased 30-day mortality in the cardiac arrest (OR 1.42, 95% CI 1.32–1.53) and sepsis subgroups (OR 1.16, 95% CI 1.11–1.22), when adjusting for age and SAPS3 (Table 2). In contrast, female sex was associated with decreased mortality in the adjusted model in the ARDS (OR 0.77, 95% CI 0.65–0.90), bacterial pneumonia (OR 0.82, 95% CI 0.74–0.92), and acute brain injury subgroups (OR 0.91, 95% CI 0.84–0.98). Subgroups where female sex was associated with increased adjusted 30-day mortality accounted for more of the total mortality than subgroups where female sex was a protective factor (Supplementary Table 4). In the adjusted model, postmenopausal females had increased 30-day mortality (OR 1.03, 95% CI 1.01–1.05) compared with males, whereas in premenopausal females no significant difference was found (Table 2). The majority (93.8%) of the 30-day mortality occurred in patients aged ≥51 yr (Supplementary Table 4).

**Table 2:** Association of female sex and 30-day mortality. ARDS, acute respiratory distress syndrome CI, confidence interval; OR, odds ratio. ∗Adjusted for age and Simplified Acute Physiology Score 3.

Subgroup	*n* (%)	Female, %	Univariable Female:male OR (95% CI)	*P*-value	Multivariable∗ Female:male OR (95% CI)	*P*-value
**All patients**	303 875 (100)	42.8	0.94 (0.92–0.95)	<0.0001	1.03 (1.01–1.05)	0.0133
**Diagnostic group**						
Cardiac arrest	16 836 (5.6)	33.7	1.45 (1.36–1.56)	<0.0001	1.42 (1.32–1.53)	<0.0001
ARDS	3338 (1.1)	35.9	0.75 (0.65–0.87)	0.0002	0.77 (0.65–0.90)	0.0015
Bacterial pneumonia	8614 (2.8)	40.6	0.80 (0.73–0.88)	<0.0001	0.82 (0.74–0.92)	0.0003
Sepsis	40 180 (13.2)	42.4	1.02 (0.98–1.06)	0.3995	1.16 (1.11–1.22)	<0.0001
Trauma	9734 (3.2)	25.2	1.33 (1.13–1.56)	0.0005	1.11 (0.91–1.36)	0.2839
Acute brain injury	19 986 (6.6)	38.8	1.02 (0.96–1.08)	0.5332	0.91 (0.84–0.98)	0.0128
**Age group**						
Premenopausal (<51 yr)	75 031 (24.7)	45.7	0.76 (0.71–0.82)	<0.0001	0.93 (0.86–1.00)	0.0580
Postmenopausal (≥51 yr)	228 844 (75.3)	41.8	0.99 (0.97–1.01)	0.2435	1.03 (1.01–1.05)	0.0102

**Fig 2:**
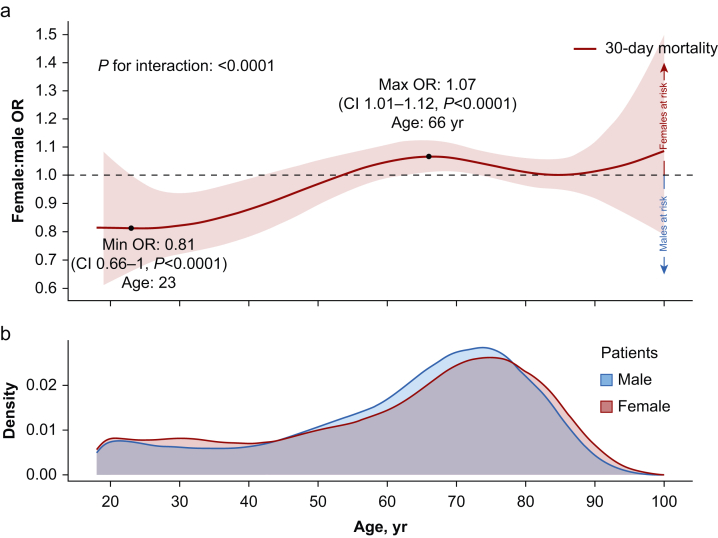
Age-specific variations in association of female sex and 30-day mortality. (a) Adjusted model including Simplified Acute Physiology Score 3 (SAPS3) and an interaction between patient sex and age with cubic splines. Solid line represents the female:male odds ratio (OR) of 30-day mortality. Shaded area is the 95% confidence interval (CI). (b) Density of all patients. Area under curve corresponds to 100%.

Figure 2a illustrates the detailed relationship between sex, age, and 30-day mortality, adjusted for SAPS3. Including the interaction term between sex and splined age improved the model fit. In the interaction model, female sex was not a constant risk factor for 30-day mortality. Instead, for younger ages, female sex was associated with reduced mortality (minimum OR 0.81, 95% CI 0.66–1.00 at 23 yr). However, with increasing age, female sex was associated with higher odds of 30-day mortality (maximum OR 1.07, 95% CI 1.01–1.12 at 66 yr). After 66 yr, the association decreased. Figure 2b describes the age distribution of ICU patients, showing that older patients comprised a larger portion of the cohort.

### Intensive care intensity

**Fig 3:**
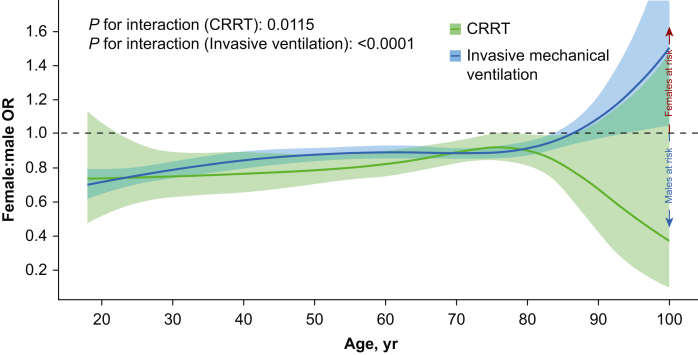
Age-specific variations in association of female sex and continuous renal replacement therapy (CRRT) or invasive ventilation. Adjusted model including Simplified Acute Physiology Score 3 (SAPS3) and an interaction between patient sex and age with cubic splines. Solid line represents the female:male odds ratio (OR). Shaded area is the 95% confidence interval.

CRRT was more common in males (5.0%) than in females (3.9%). Similarly, males were more likely than females to be treated with invasive ventilation (36.1% *vs* 31.4%; Table 1). When adjusting for age and SAPS3, female sex was less associated with CRRT treatment in the overall cohort (OR 0.82, 95% CI 0.79–0.85) and in all diagnostic subgroups (Table 3). Similarly, female sex was less associated with invasive ventilation in the overall cohort (OR 0.86, 95% CI 0.84–0.87) and in all diagnostic subgroups except for pneumonia, sepsis, and acute brain injury. In acute brain injury, females had higher odds of undergoing invasive ventilation than males. Figure 3 describes the relationship between female sex, age, and interventions in the ICU adjusted for SAPS3. The interaction model was not a significantly better fit for the CRRT model and in invasive ventilation the association was visually rather constant.

**Table 3:** Association of female sex and continuous renal replacement therapy (CRRT) or invasive mechanical ventilation. ARDS, acute respiratory distress syndrome; CI, confidence interval; OR, odds ratio. ∗Adjusted for Simplified Acute Physiology Score 3 and age.

Subgroup	*n*	Female, %	Univariable Female:male OR (95% CI)	*P*-value	Multivariable∗ Female:male OR (95% CI)	*P*-value
**Outcome: CRRT**						
**All patients**	303 875	42.8	0.76 (0.73–0.79)	<0.0001	0.82 (0.79–0.85)	<0.0001
**Diagnostic group**						
Cardiac arrest	16 836	33.7	0.76 (0.65–0.89)	0.0007	0.72 (0.61–0.85)	0.0001
ARDS	3338	35.9	0.60 (0.47–0.76)	<0.0001	0.57 (0.44–0.72)	<0.0001
Pneumonia	8614	40.6	0.76 (0.62–0.92)	0.0057	0.81 (0.66–0.99)	0.0458
Sepsis	40 180	42.4	0.85 (0.80–0.90)	<0.0001	0.89 (0.84–0.94)	0.0001
Trauma	9734	25.2	0.49 (0.29–0.77)	0.0037	0.46 (0.27–0.73)	0.0020
Acute brain injury	19 986	38.8	0.54 (0.31–0.92)	0.0271	0.55 (0.31–0.93)	0.0307
**Age group**						
Premenopausal (<51 yr)	75 031	45.7	0.68 (0.62–0.74)	<0.0001	0.77 (0.70–0.85)	<0.0001
Postmenopausal (≥51 yr)	228 844	41.8	0.79 (0.76–0.82)	<0.0001	0.85 (0.82–0.89)	<0.0001

**Outcome: invasive ventilation**						
**All patients**	303 875	42.8	0.81 (0.80–0.82)	<0.0001	0.86 (0.84–0.87)	<0.0001
**Diagnostic group**						
Cardiac arrest	16 836	33.7	0.88 (0.80–0.96)	0.0030	0.84 (0.76–0.92)	0.0002
ARDS	3338	35.9	0.89 (0.76–1.04)	0.1302	0.84 (0.72–0.98)	0.0279
Pneumonia	8614	40.6	0.87 (0.80–0.96)	0.0030	0.92 (0.83–1.01)	0.0649
Sepsis	40 180	42.4	0.94 (0.90–0.97)	0.0014	0.97 (0.93–1.01)	0.1139
Trauma	9734	25.2	0.85 (0.77–0.93)	0.0006	0.86 (0.76–0.96)	0.0091
Acute brain injury	19 986	38.8	1.08 (1.02–1.14)	0.0070	1.09 (1.02–1.17)	0.0158
**Age group**						
Premenopausal (<51 yr)	75 031	45.7	0.76 (0.74–0.79)	<0.0001	0.81 (0.78–0.84)	<0.0001
Postmenopausal (≥51 yr)	228 844	41.8	0.84 (0.82–0.85)	<0.0001	0.89 (0.88–0.91)	<0.0001

## Discussion

This nationwide cohort study investigating sex differences in the ICU found that crude mortality was higher in male patients, but when comparing female and male patients with the same age and SAPS3, female patients had increased odds of mortality. These sex differences were further nuanced by age: female sex had an increased association to mortality in older patients, but the opposite association in younger patients. While most of the mortality could be attributed to the older part of the cohort where female patients had an increased risk, death was rather uncommon in the younger part where male patients appeared to be at risk. Nevertheless, despite the small absolute risk, young patients have a longer life expectancy and consequently lost more life-years.

### Comparison with the literature

The higher crude mortality observed in male patients could be explained by greater illness severity at ICU admission, as increased mortality in male patients was not present after adjustment for age and SAPS3. Given that SAPS3 is a well-established predictor of mortality in the ICU,^17^ it is expected that patients with higher scores face increased risk of death. In contrast to our results showing higher SAPS3 in male patients, most studies have previously reported higher SAPS3 scores in female patients.^2^^,^^18^

When comparing male and female patients at equal age and SAPS3, in the adjusted models, female patients instead had a small but significant increased mortality, meaning that despite having the same predicted mortality at admission females were at higher risk. Previous findings on sex differences in adjusted mortality have been inconsistent, with some studies reporting higher adjusted mortality in females than in males,^12^ and others finding no significant difference.^1^ A large meta-analysis,^19^ which included 21 studies and 505 138 participants, indicated that female patients had increased risk-adjusted mortality at ICU discharge and 1 yr after admission. However, these results were not significant when removing studies with high risk of bias.

Age variations may explain conflicting results in previous studies as our spline interaction model revealed fluctuating associations between sex and 30-day mortality across ages. Previous studies have often compared only two age groups. For example, Mahmood and colleagues^7^ observed reduced ICU mortality in female patients aged <50 yr (OR 0.83, 95% CI 0.76–0.91), whereas Fowler and colleagues^2^ reported increased ICU mortality in female patients aged >50 yr (OR 1.20, 95% CI 1.10–1.31). In contrast, Samuelsson and colleagues^1^ found no sex difference in mortality in patients aged <45 or ≥45 yr, which might be explained by a smaller sample size. Although previous studies, and ours, have detected only minor differences in ORs, even small variations can yield clinically significant effects given the inherently high mortality rates in the ICU.

Higher care intensity in males was present in both crude and adjusted numbers. Although the consensus is that most interventions are more common in male patients in the ICU,^6^ there are few prior studies investigating this while stratifying by age. Unlike age-related mortality changes, sex differences in CRRT and invasive ventilation showed little variation by age in this study, suggesting that excess mortality is unlikely to result from disparities in CRRT or invasive ventilation between sexes.

### Potential mechanisms

There is no established rationale for expecting differences in mortality or treatment between male and female patients at similar illness severity in the ICU, but several biological, clinical, and social factors might influence our results. We present five hypothetical mechanisms of our findings.

#### Simplified Acute Physiology Score 3 calibration

The comparison of male and female patients at the same SAPS3 score assumes that SAPS3 performance is independent of sex, but if female patients are more severely ill than male patients with the same SAPS3 score, this might affect the adjusted results. SAPS3 includes a range of variables, from comorbidities to illness severity at admission, but is not separately calibrated for male and female patients despite including variables with known sex differences, such as creatinine concentrations.^20^ Although no study has examined these properties regarding SAPS3, it has previously been suggested that the Sequential Organ Failure Assessment score does not fit male and female patients equally well.^21^^,^^22^ However, although sex differences in SAPS3 performance could potentially explain higher adjusted mortality in female patients, the same assumption would instead further increase differences in care intensity. As male patients received more intense treatment at the same SAPS3 score in our study, this would contradict the hypothesis that SAPS3 underestimates illness severity in female patients. Furthermore, as sex differences in mortality varied by age, SAPS3 performance would also need to be age-varying.

#### Hormonal influence

As oestrogen concentrations differ between males and females and vary by age, hormones could potentially explain the sex differences in ICU mortality found in this study. Protective effects of oestrogen could correspond to the minimum risk in female patients compared with male patients at 23 yr, when oestrogen concentrations are generally high in females, and the maximum risk at 66 yr, when oestrogen concentrations are low. Protective effects of oestrogen have been demonstrated in animal models,^9^ but this has not been confirmed in clinical studies.^23^^,^^24^ Despite previous contradictory findings, new research initiatives are currently being conducted in this area. A recent investigation of the immune system in transgender men undergoing gender-affirming testosterone treatment has revealed that testosterone therapy modifies monocytic and type-1 interferon responses.^25^ This suggests that sex hormones, independent of genetic composition, may influence factors related to disease resistance. Moreover, a preliminary study investigating single-dose oestrogen therapy in trauma haemorrhagic shock showed a reduction in serum cytokines and tendencies towards favourable outcomes, but the study was too small to reach significant results.^26^

#### Admission patterns

Admission patterns to the ICU might differ between male and female patients, resulting in a selection bias, potentially affecting our results. Prior research has indicated that female patients are less likely to be admitted to the ICU than male patients.^4^ In a comprehensive study of sex differences in admissions, Modra and colleagues^19^ reported that patients aged 60–69 yr had the lowest percentage of female patients and the lowest association between female sex and ICU admission. This is consistent with our result with a higher proportion of male patients, most pronounced in patients >51 yr. If female patients who may require intensive care are found ineligible for admission, this could contribute to the higher crude mortality rate and elevated SAPS3 scores observed in male patients.

#### Limitations of life-sustaining treatment

Both treatment intensity and mortality in the ICU could be affected by limitations of life-sustaining treatment. A meta-analysis found that female sex was associated with increased likelihood of limitations of life-sustaining treatment.^27^ A contributing factor to this association could be the higher proportion of older women who are widowed, as marital status is associated with limitations of life-sustaining treatment.^28^ In 2014, the proportion of widows in Sweden was 6.8% for women aged 65 yr and 41.5% for women aged 80 yr.^29^ In contrast, the proportion of widowers was 2.7% for men aged 65 yr and 14.0% for men aged 80 yr. If older female patients are more often addressed with life-sustaining treatment limitations than male patients at the same SAPS3 score, this may explain the sex differences in adjusted mortality. However, as limitations are uncommon in younger ages, this would most likely not explain the fact that less intense treatment in female patients was constant over most ages.

#### Implicit bias

Implicit bias, referring to unconscious attitudes or stereotypes that influence judgement and behaviour, has been shown to influence decision-making and patient management in various medical fields.^30^ An implicit sex bias in ICU admission and care could contribute to our results, potentially leading to less intense treatment in female patients and increased mortality among older female and younger male patients. However, implicit bias is challenging to investigate, and previous studies have not identified bias among intensivists against female patients in ICU admission surveys.^31^ Modra and colleagues^8^ proposed that sex-based differences in ICU mortality risk depend on the proportion of male and female patients within specific diagnostic groups. According to this hypothesis, in diagnoses with a lower proportion of female patients compared with the overall ICU population, female sex is more strongly associated with mortality. Conversely, in diagnoses with a lower proportion of male patients, male sex would be the risk factor. Such patterns may originate from implicit bias. Although the sepsis and cardiac arrest subgroups in our study align with this hypothesis, the ARDS and bacterial pneumonia groups contradict it. Interestingly, age-related patterns support the hypothesis, as both older female patients and younger male patients, being lower-proportion groups in this study, exhibit a higher risk.

### Future research

As this study was not designed to investigate the mechanisms of sex differences in treatment and mortality in the ICU, our explanations for our findings remain speculative. There is a need to explore sex-specific performance of scoring systems in the ICU, and we also encourage future studies to investigate sex differences in life-sustaining treatment limitations and their potential impact on sex differences in the ICU care and outcomes. Furthermore, little is known about effects of potential implicit bias in the ICU.

### Strengths and limitations

We used a large national registry that includes individual patient data from all 81 ICUs in Sweden to investigate sex differences, combining both treatment and mortality across the entire adult ICU population. The Registry collects data prospectively for quality surveillance purposes, which counteracts bias in relation to this study. We described the age-varying impact on sex differences in the ICU continuously rather than in larger age groups.

The registry-based method limits the selection of variables, which means that important factors such as socioeconomic status and ethnicity were lacking. Using only comorbidity data from SAPS3 may potentially contribute to unmeasured bias and we cannot fully dismiss that results derive from this. As SAPS3 is used as a proxy for illness severity in the adjusted model, the calibration of the individual coefficients of SAPS3 may affect the results. The individual calibration of coefficients used in SAPS3 has not been validated or recalibrated since its development. Furthermore, as decisions to limit life-sustaining treatment might vary between male and female patients, the lack of this variable could influence the results.

### Conclusions

These findings indicate that although male patients constitute a larger proportion of patients in the ICU and are more frequently subjected to advanced treatments, female sex is associated with increased adjusted mortality. However, this is nuanced by age: female sex emerges as a risk factor for mortality at older ages, whereas at younger ages the effect is reversed. This underscores the complexity and necessity of considering age when evaluating sex-based differences in ICU outcomes.

## Authors' contributions

Had full access to the data, ensuring its integrity, accuracy, and the decision to submit: EH, JE, EL

Concept and design: EH, JE, EL

Data acquisition, analysis, or interpretation: all authors

Manuscript drafting: EH, EL

Critical revision of the manuscript: all authors

Statistical analysis: EH, JE, EL

Administrative, technical, or material support: JE, LE, EL

Supervision: JE, EL

Approval of the final manuscript: all authors.

## Data availability statement

The dataset analysed during the current study is not publicly available or available for sharing, as it contains personal data. On reasonable request, data could be made available after permission from the Swedish Ethical Review Authority and Swedish Intensive Care Registry.

## Declaration of generative AI and AI-assisted technologies in the writing process

During the preparation of this work, the authors used Microsoft Copilot in order to review the text and improve its readability. After using this tool, the authors reviewed and edited the content as needed and take full responsibility for the content of the publication.

## Funding

Open access funding was provided by Karolinska Institutet and Region Stockholm. They had no role in the study's design, conduct, data analysis, or manuscript preparation and submission.

## Declaration of interest

The authors declare that they have no competing interests.
