# Supplementary material for: Age-related sex differences in intensive care treatment and outcomes: a nationwide cohort study
Source: Br J Anaesth. 2025 Aug 29;136(4):1217–25. doi: 10.1016/j.bja.2025.07.044 (PMC13014495; doi:10.1016/j.bja.2025.07.044)
Supplement: Multimedia component 1 [file mmc1.docx]

**Supplementary Table 1.** **Association of female sex and 30-day mortality, excluding patients 2020-03-10 to 2021-09-27 (COVID-19 period).** Univariable and multivariable logistic regression. All models adjusted for SAPS3 and age. Female:male OR presented for all admissions. Stratified analyses presenting female:male OR for diagnostic subgroups and age groups separately.

| **Subgroup** | **n** | **Univariable Female:male**  **OR (95% CI)** | **Multivariable^a^**  **Female:male**  **OR (95% CI)** |
| --- | --- | --- | --- |
| **All admissions** | 268 564 | 0.95 (0.93-0.96)*** | 1.05 (1.02-1.07)*** |
| **Diagnostic group** |  |  |  |
| Cardiac arrest | 14 955 | 1.45 (1.36-1.56)*** | 1.41 (1.30-1.52)*** |
| ARDS | 2 458 | 0.74 (0.63-0.87)*** | 0.79 (0.66-0.95)* |
| Bacterial pneumonia | 8 122 | 0.81 (0.73-0.90)*** | 0.83 (0.74-0.92)*** |
| Sepsis | 35 406 | 1.02 (0.97-1.07) | 1.19 (1.13-1.25)*** |
| Trauma | 8 752 | 1.27 (1.07-1.50)** | 1.05 (0.85-1.30) |
| Acute brain injury | 17 628 | 1.04 (0.97-1.10) | 0.92 (0.84-0.99)* |
| **Age group** |  |  |  |
| Premenopausal (<51 years) | 66 871 | 0.75 (0.70-0.81)*** | 0.93 (0.85-1.01) |
| Postmenopausal (≥51 years) | 201 693 | 1.00 (0.98-1.02) | 1.05 (1.03-1.08)*** |

a adjusted for SAPS3 and age, * p<0.05, ** p<0.01, *** p<0.001
