# Supplementary material for: Age-related sex differences in intensive care treatment and outcomes: a nationwide cohort study
Source: Br J Anaesth. 2025 Aug 29;136(4):1217–25. doi: 10.1016/j.bja.2025.07.044 (PMC13014495; doi:10.1016/j.bja.2025.07.044)
Supplement: Multimedia component 2 [file mmc2.docx]

**Supplementary Table 2.** **Association of female sex and 30-day mortality, mixed model with region as random effect.** Univariable and multivariable logistic regression. All models adjusted for SAPS3 and age. Female:male OR presented for all admissions. Stratified analyses presenting female:male OR for diagnostic subgroups and age groups separately.

| **Subgroup** | **n** | **Univariable Female:male**  **OR (95% CI)** | **Multivariable^a^**  **Female:male**  **OR (95% CI)** |
| --- | --- | --- | --- |
| **All admissions** | 303 875 | 0.94 (0.92-0.96)*** | 1.03 (1.01-1.05)* |
| **Diagnostic group** |  |  |  |
| Cardiac arrest | 16 836 | 1.43 (1.33-1.53)*** | 1.41 (1.30-1.52)*** |
| ARDS | 3 338 | 0.74 (0.64-0.86)*** | 0.76 (0.65-0.90)** |
| Bacterial pneumonia | 8 614 | 0.80 (0.72-0.88)*** | 0.82 (0.74-0.92)*** |
| Sepsis | 40 180 | 1.02 (0.97-1.06) | 1.16 (1.11-1.22)*** |
| Trauma | 9 734 | 1.34 (1.14-1.58)*** | 1.12 (0.92-1.36) |
| Acute brain injury | 19 986 | 1.06 (1.00-1.13) | 0.94 (0.87-1.02 |
| **Age group** |  |  |  |
| Premenopausal (<51 years) | 75 031 | 0.79 (0.74-0.84)*** | 0.94 (0.86-1.01) |
| Postmenopausal (≥51 years) | 228 844 | 0.99 (0.97-1.01) | 1.03 (1.01-1.05)* |

a adjusted for SAPS3, age and the random effect of region, * p<0.05, ** p<0.01, *** p<0.001
