# Supplementary material for: Age-related sex differences in intensive care treatment and outcomes: a nationwide cohort study
Source: Br J Anaesth. 2025 Aug 29;136(4):1217–25. doi: 10.1016/j.bja.2025.07.044 (PMC13014495; doi:10.1016/j.bja.2025.07.044)
Supplement: Multimedia component 3 [file mmc3.docx]

**Supplementary Table 3.** **Association of female sex and 30-day mortality, multivariable model only adjusting for age.** Univariable and multivariable logistic regression. Multivariable models adjusted for age. Female:male OR presented for all admissions. Stratified analyses presenting female:male OR for diagnostic subgroups and age groups separately.

| **Subgroup** | **n** | **Univariable Female:male**  **OR (95% CI)** | **Multivariable^a^**  **Female:male**  **OR (95% CI)** |
| --- | --- | --- | --- |
| **All admissions** | 303 875 | 0.94 (0.92-0.96)*** | 0.91(0.89-0.93)*** |
| **Diagnostic group** |  |  |  |
| Cardiac arrest | 16 836 | 1.43 (1.33-1.53)*** | 1.44 (1.35-1.54)*** |
| ARDS | 3 338 | 0.74 (0.64-0.86)*** | 0.81 (0.69-0.95)** |
| Bacterial pneumonia | 8 614 | 0.80 (0.72-0.88)*** | 0.79 (0.71-0.87)*** |
| Sepsis | 40 180 | 1.02 (0.97-1.06) | 1.06 (1.02-1.11)** |
| Trauma | 9 734 | 1.34 (1.14-1.58)*** | 1.09 (0.92-1.29) |
| Acute brain injury | 19 986 | 1.06 (1.00-1.13) | 0.96 (0.91-1.03) |
| **Age group** |  |  |  |
| Premenopausal (<51 years) | 75 031 | 0.79 (0.74-0.84)*** | 0.79 (0.74-0.84)*** |
| Postmenopausal (≥51 years) | 228 844 | 0.99 (0.97-1.01) | 0.93 (0.91-0.94)*** |

a adjusted for age, * p<0.05, ** p<0.01, *** p<0.001
