# Supplementary material for: Age-related sex differences in intensive care treatment and outcomes: a nationwide cohort study
Source: Br J Anaesth. 2025 Aug 29;136(4):1217–25. doi: 10.1016/j.bja.2025.07.044 (PMC13014495; doi:10.1016/j.bja.2025.07.044)
Supplement: Multimedia component 4 [file mmc4.docx]

**Supplementary Table 4. 30-day mortality in diagnostic subgroups.**

No. and percentage of total 30-day mortality in the cohort.

|  | N (%) | 30-day mortality( % of total 30-day mortality) |
| --- | --- | --- |
| **All admissions** | 303 875 (100%) | 61 199 (100%) |
| **Diagnostic group** |  |  |
| Cardiac arrest | 16 836 (5.5%) | 10 573 (17.3%) |
| ARDS | 3 338 (1.1%) | 1 331 (2.2%) |
| Bacterial pneumonia | 8 614 (2.8%) | 2 334 (3.8%) |
| Sepsis | 40 180 (13.2%) | 12 234 (20.0%) |
| Trauma | 9 734 (3.2%) | 778 (1.3%) |
| Acute brain injury | 19 986 (6.6%) | 6 186 (10.1%) |
| **Age group** |  |  |
| Premenopausal (<51 years) | 75 031 (24.7%) | 3 814 (6.2%) |
| Postmenopausal (≥51 years) | 228 844 (75.3%) | 57 385 (93.8%) |
