# Supplementary material for: Age-related sex differences in intensive care treatment and outcomes: a nationwide cohort study
Source: Br J Anaesth. 2025 Aug 29;136(4):1217–25. doi: 10.1016/j.bja.2025.07.044 (PMC13014495; doi:10.1016/j.bja.2025.07.044)
Supplement: Multimedia component 5 [file mmc5.docx]

**Supplementary Table 5. Diagnostic subgroups based on ICD-10 code**

**Cardiac arrest**

Cardiac arrest I46

Cardiac arrest with successful resuscitation I46.0

Sudden cardiac death I46.1

Cardiac arrest, unspecified I46.9

**ARDS**

ARDS, unspecifed J80.9X

Mild ARDS

PaO2/FiO2 26-40 and PEEP 5 or more J80.9A

Moderate ARDS

PaO2/FiO2 13-25 and PEEP 5 or more J80.9B

Severe ARDS

PaO2/FiO2 less than 13 and PEEP 5 or more J80.9C

**Bacterial pneumonia**

Pneumonia due to Streptococcus pneumoniae J13

Pneumonia due to Haemophilus influenzae J14

Pneumonia due to Klebsiella pneumoniae J15.0

Pneumonia due to Pseudomonas J15.1

Pneumonia due to Staphylococcus J15.2

Pneumonia due to Staphylococcus, group B J15.3

Pneumonia due to other Streptococci J15.4

Pneumonia due to Escherichia coli J15.5

Pneumonia due to other aerobic Gram-negative bacteria J15.6

Pneumonia due to Mycoplasma pneumoniae J15.7

Other bacterial pneumonia J15.8

Bacterial pneumonia, unspecified J15.9

**Sepsis**

Septic chock according to Sepsis 3 criteria R57.2

Sepsis according to Sepsis 3 criteria R65.1

**Trauma**

Unspecified multiple trauma T07.9

**Acute brain injury**

Subarachnoid hemorrhage (non-traumatic) I60.0→I60.9

Intracerebral supratentorial hemorrhage I61.0→I61.2

Intracerebral hemorrhage in brain stem I61.3

Intracerebral hemorrhage in cerebellum I61.4

Intraventricular hemorrhage I61.5

Intracerebral hemorrhage, unspecified I61.6→I61.9

Subdural hemorrhage (non-traumatic) I62.0

Epidural hemorrhage (non-traumatic) I62.1

Intracranial hemorrhage (non-traumatic, unspecified) I62.9

Cerebral infarction I63.0→I63.5, I63.9

Cerebral sinus thrombosis I63.6

Cerebrovascular disorder, other I64.9→I69.8

Injuries to the head S00.0→S00.9, S03.0→S05.9, S07.0→S09.9

Open wound of the head S01.0→S01.9

Fracture of base of skull S02.1→S02.11

Fracture involving skull and facial bones S02.0→S02.01, S02.2→S02.91

Concussion S06.0→S06.01

Brain edema, traumatic S06.1→S06.11

DAI. Diffuse contusion / laceration S06.2→S06.21

Focal cerebral contusion S06.3→S06.31

Epidural hemorrhage, traumatic S06.4→S06.41

Subdural hemorrhage, traumatic S06.5→S06.51

Subarachnoid hemorrhage, traumatic S06.6→S06.61

Intracranial injury with prolonged coma S06.7→S06.71

Other intracranial injury, unspeicified S06.8→S06.91
